# The current status of interventional radiology in the undergraduate medical curriculum and the way forward

**DOI:** 10.1259/bjr.20220197

**Published:** 2022-11-21

**Authors:** Filzah Hanis Osman, Jasmine Sze Ern Koe, Elisa Shi Wei Lau, Dhikshitha Nagaraj, Helen Hoi-Lam Ng, Alexander Ng, Aqua Asif, Raman Uberoi, Raghuram Lakshminarayan, Vinson Wai-Shun Chan, Tze Min Wah

**Affiliations:** School of Medicine, Faculty of Medicine and Health, University of Leeds, Leeds, United Kingdom; School of Medicine, Faculty of Medicine and Health, University of Leeds, Leeds, United Kingdom; School of Medicine, Faculty of Medicine and Health, University of Leeds, Leeds, United Kingdom; School of Medicine, Faculty of Medicine and Health, University of Leeds, Leeds, United Kingdom; School of Medicine, Faculty of Medicine and Health, University of Leeds, Leeds, United Kingdom; School of Medicine, Faculty of Medicine and Health, University of Leeds, Leeds, United Kingdom; School of Medicine, Faculty of Medicine and Health, University of Leeds, Leeds, United Kingdom; Department of Radiology, John Radcliffe Hospital, Oxford, United Kingdom; Department of Vascular Radiology, Hull University Teaching Hospitals NHS Trust, Hull, United Kingdom; School of Medicine, Faculty of Medicine and Health, University of Leeds, Leeds, United Kingdom; Division of Diagnostic and Interventional Radiology, Institute of Oncology, St. James’s University Hospital, Leeds, United Kingdom

## Abstract

Interventional radiology (IR) is underrepresented in undergraduate medical school curricula. Despite the introduction of a suggested undergraduate curriculum for IR by the British Society of Interventional Radiology (BSIR), current evidence suggests there is inadequate knowledge and awareness of IR amongst medical students. As a result of this, there is a lack of visibility of the subspeciality amongst medical students and junior doctors contributing to the shortage of IR trainees resulting in an IR workforce crisis in the UK. The uptake of the proposed undergraduate IR curriculum remains unclear, highlighting the need for a thorough audit and improvement of IR teaching in undergraduate medical education. In this commentary, we discuss the importance of including IR in the undergraduate curriculum, the evidence surrounding undergraduate IR education, the reasons for the potential lack of interest in IR from medical students and future steps to ensure optimal IR exposure in undergraduate medical school curricula.

Interventional radiology (IR) worldwide has become one of the key medical subspecialties delivering minimally invasive treatments to a wide range of patients, transforming the way we manage patients in the twenty-first century. The concept of using image-guided minimally-invasive techniques, to treat patients, was first developed by Charles T. Dotter, whose main scope of work surrounded the innovative use of wires and catheters for diagnosis and treatment in replacement of the scalpel.^1^ Dr Dotter performed the world’s first successful percutaneous transluminal angioplasty in 1963, on an 82-year-old female who had a critically ischaemic leg. The patient, who had refused leg amputation, eventually walked out of the hospital on both feet owing to Dr Dotter’s technique of using a guide wire and catheter to dilate the stenosed superficial femoral artery. The discovery of IR techniques has opened doors to a plethora of minimally invasive procedures, revolutionising the treatment of many malignant and benign conditions, in both elective and emergency settings. This ranges from life-saving procedures such as embolisation therapy to control gastrointestinal bleeding and trauma bleeding, repair of abdominal aorta aneurysms, and cancer treatments, to those that enhance quality of life for people with chronic conditions such as stent insertions in peripheral arterial disease.

Unsurprisingly, the popularity of IR procedures has increased in recent years, where IR procedures are being advocated due to their improved surgical and recovery outcomes. When compared to their surgical counterparts, IR procedures have demonstrated advantages in reducing length of hospital stays, decreasing morbidity and mortality, improving survival outcomes, and reducing post-operative complications.^2–5^ Hence, there has been an ever-increasing role for IR treatments, sometimes replacing or complementing established surgical techniques.

However, there is a significant shortage of interventional radiologists in the UK, denying patients the opportunity to benefit from these new and innovative techniques.^6^ The Royal College of Radiologists (RCR) guidelines recommend that services consisting of six or more interventional radiologists will usually be able to provide a reliable twenty-four-hour IR service/million population. However, RCR’s latest workforce census reveals that half the trusts in the UK (47%) admitted to not having enough manpower to provide timely IR access, resulting in patients potentially missing out on life-saving procedures, such as the drainage of obstructions from the biliary and urinary system, endovascular treatment of gastrointestinal bleeding and aneurysms, removal of stroke clots and embolisation of cancer tumours.^6^ It is further reported that an additional of 364 IR consultants are required in order to deliver out-of-hours services, as a consequence of an insufficient number of doctors being trained in this field.^6^,^7^

Figure 1 shows the current IR training pathway, recreated from RCR’s latest version of the IR Specialty Training Curriculum.^8^ Following graduation from medical school, junior doctors are first required to complete their two-year foundation programme before applying for Core Radiology training. Trainees are then required to train for a period of three years in Core Clinical Radiology training and the remaining three in IR Specialist training. A pilot for specialty training year 1 (ST1) in clinical radiology with focus in IR is undergoing, and outcomes are eagerly awaited. Following the completion of the six years training programme, trainees will be awarded with a Certification of Completion of Training (CCT) in clinical radiology, with interventional radiology as a subspecialisation.

**Figure 1.**
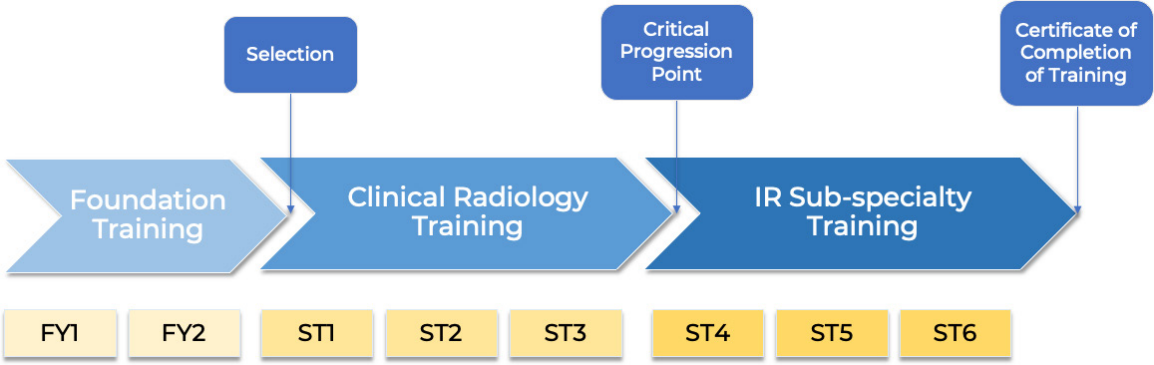
Current training pathway for Interventional Radiology ^8^

In light of IR’s workforce crisis, it would be worthwhile to explore the causes behind the low uptake of trainees. Several studies, targeted at junior doctors and medical students, have developed questionnaires to better understand factors influencing future career choices.^9,10^ A recent study by El Farargy et al which surveyed 79 Foundation trainees in the UK, found that 75% of the respondents would not consider a career in IR, primarily due to a lack of general interest in radiology.^9^ With the lack of interest, coupled with the lack of information on the career option, junior doctors are disinclined to pursue IR as a career. When the Foundation trainees were asked to rate possible ways of raising awareness about IR, the most popular method was deemed to be elective placements and teaching during Foundation years, followed by lectures in medical schools.^9^ Similarly, a study on delegates consisting of medical students and junior doctors at a UK-based online IR symposium, further suggests that more dedicated IR clinic time within the curriculum would be helpful in boosting the exposure of medical students to IR.^10^

Hence, medical schools play a key role in equipping medical students with sufficient knowledge on IR, before they go on to be Foundation trainees. Another study exploring the factors influencing junior doctors and medical students’ willingness to pursue IR as a career, found that involvement in IR clinical activities both in the undergraduate and extracurricular settings were key contributors.^11^ While the authors highlighted that an early interest in IR during medical school was not critical in pursuing IR in the future, there is still a dire need for better representation of IR in the undergraduate curriculum.

Therefore, a national curriculum is essential in establishing consistent undergraduate IR teaching across medical schools. Following the acknowledgment of IR’s subspecialty status in the UK in 2010, the British Society of Interventional Radiology (BSIR) published an undergraduate curriculum highlighting the need for integrated teaching in medical schools, with the aim of providing guidance to prepare medical students for their roles as junior doctors, as well as to stimulate interest in a career in IR.^12^ The suggested curriculum outlines the principles behind basic and common procedures that Foundation doctors would expect to encounter, including the relevant anatomy and physiology that ought to be covered during pre-clinical basic science teaching. Other aspects of the curriculum include the basic principles of IR techniques, as well as issues pertaining to patient preparation, consent and common complications of IR procedures. These are expected to be delivered throughout undergraduate modules and clinical attachments, be it fully or partially. It is also highlighted that since there are no existing IR foundation competencies expected of a newly qualified doctor by the General Medical Council (GMC), it is therefore important that the IR curriculum is consolidated through the inclusion of IR-related topics in undergraduate clinical examinations and OSCE exams. Likewise, RCR’s undergraduate radiology curriculum also suggests the incorporation IR teaching within the undergraduate curriculum, with the aim of helping students understand the therapeutic options that IR can offer to patients in acute and chronic settings.^13^ The learning outcomes are outlined by the roles of both diagnostic imaging and IR in the investigation and management of common clinical scenarios.

However, whilst there are recommendations in place for medical schools to incorporate the undergraduate IR curriculum into their own as part of modules and clinical attachments, there are still reports of a lack of exposure to IR in the current teachings of medical schools. A recent study of two English medical schools reported that 81.4% of students have not received formal teaching in IR and over 70% wanted more exposure in IR.^14^ Consequently, this affects the chances of IR being pursued as a career path by medical students upon graduation.^14^ Whilst previous studies have assessed the level of knowledge of IR amongst medical students, there is a need for a formal evaluation of the extent of the recommended BSIR undergraduate curriculum being incorporated into UK medical schools, as the uptake of the proposed curriculum has been unclear.

In view of this, the nationwide ELIXIR study^15^ – *An Evaluation of LearnIng and eXposure in the undergraduate Interventional Radiology curriculum,* has been launched with the aim of evaluating the current state of IR teaching in the undergraduate curriculum in medical schools across the UK. Based on the distribution of an online survey to final year medical students in the UK, it also aims to evaluate the overall degree of awareness of IR amongst medical students. We await the results of the study, which will be helpful in identifying areas of improvement in medical students’ experiences with IR.

IR has played an increasingly important role in patient care, and it is important that current and future generations have the opportunity to become interventional radiologists to support these important therapies. While the minimally invasive nature of IR has proven to be beneficial in improving patient care as well as ensuring patient safety, the dire workforce crisis faced by IR units may expose patients to increased risk due to the lack of availability of a local 24/7 service. Action by the medical community needs to be taken immediately, and what better way to start than to inspire future interventional radiologists in medical schools?
